# Two protein/protein interaction assays in one go

**DOI:** 10.15252/msb.20188485

**Published:** 2018-07-18

**Authors:** Mikko Taipale

**Affiliations:** ^1^ Department of Molecular Genetics Donnelly Centre for Cellular and Biomolecular Research University of Toronto Toronto ON Canada

**Keywords:** Genome-Scale & Integrative Biology, Methods & Resources, Network Biology

## Abstract

Discovering and characterizing protein–protein interactions (PPIs) that contribute to cellular homeostasis, development, and disease is a key priority in proteomics. Numerous assays for protein–protein interactions have been developed, but each one comes with its own strengths, weaknesses, and false‐positive/false‐negative rates. Therefore, it seems rather intuitive that combining multiple assays is beneficial for robust and reliable discovery of interactions. Along those lines, in their recent study, Wanker and colleagues (Trepte *et al*, 2018) combined two complementary and quantitative interaction assays in one pot. One assay is luminescence‐based and depends on protein proximity in living cells, while the other relies on formation of more stable complexes detected by co‐precipitation with a luminescence‐based readout, which facilitates confident identification and quantitation of interactions in high throughput.

Protein–protein interactions (PPIs) are everywhere. It would be difficult—if not impossible—to name a cellular process that is not regulated by PPIs. Yet, nearly two decades after the protein coding complement of the human genome was established, our view of the protein interaction network of human cells is far from complete. It is certainly not for the lack of trying, as anyone who wants to find interacting partners of their favorite protein can pick an assay based on cost, detection method, scalability, or model system (Snider *et al*, 2015). The methods are as diverse as their acronyms; some are based on genetic selection (Y2H: yeast two‐hybrid) or reporter genes (MaMTH: mammalian membrane two‐hybrid; MAPPIT: mammalian protein/protein interaction trap; and KISS: kinase substrate sensor), others on protein complementation (BiFC: bimolecular fluorescence complementation; NanoBit: NanoLuc complementation assay; and DHFR‐PCA: dihydrofolate reductase protein‐fragment complementation assay) or co‐purification (AP‐MS: affinity purification–mass spectrometry; LUMIER (with BACON): luminescence‐based mammalian interactome assay (with bait control); and NAPPA: nucleic‐acid programmable protein array), yet others on protein proximity rather than direct interaction (BRET: bioluminescence resonance energy transfer; FRET: fluorescence/Foerster resonance energy transfer; and BioID: proximity‐dependent biotin identification).

So why do not we have one perfect assay that would make all others obsolete? The problem is the sheer diversity of PPIs. At the cellular level, PPIs occur in all compartments, with their characteristic pH, redox environment, lipid constitution, and posttranslational modifications. Biophysically, the affinities of known PPIs range from femtomolar (Vicentini *et al*, 2002) to millimolar (Garcia *et al*, 1996), corresponding to a staggering 12 orders of magnitude. Adding complexity, many interactions are regulated by e.g. posttranslational modifications, conformational changes, or proteolytic cleavage, which can be difficult to control experimentally. As a consequence, every single protein and all of its interactions react differently to experimental parameters, and it is impossible to optimize one assay simultaneously for all interactions and all parameters. Ideally, one would then simultaneously use multiple methods to study PPIs.

This is where the work of Trepte *et al* (2018) comes in. They have developed LuTHy, a novel method which combines co‐purification and proximity methods in one system to assay binary PPIs (Fig 1). In their assay, one protein is fused to NanoLuc luciferase and the other protein to protein A (PA) and mCitrine, a yellow variant of green fluorescent protein. After introducing the fusion proteins to mammalian cells, BRET between the two proteins is measured in living cells. BRET occurs only if the two proteins are in close proximity (within 10 nm, or the diameter of an average globular protein), in which case photons emitted by the NanoLuc donor fused to one protein can excite the mCitrine acceptor fused to the other. After this step, the PA‐mCitrine fusion protein is adsorbed on a plate coated with immunoglobulin IgG to which PA binds with high affinity. If the two proteins form a stable complex, luminescence signal can be observed after washing off nonspecifically binding proteins.

**Figure 1:**
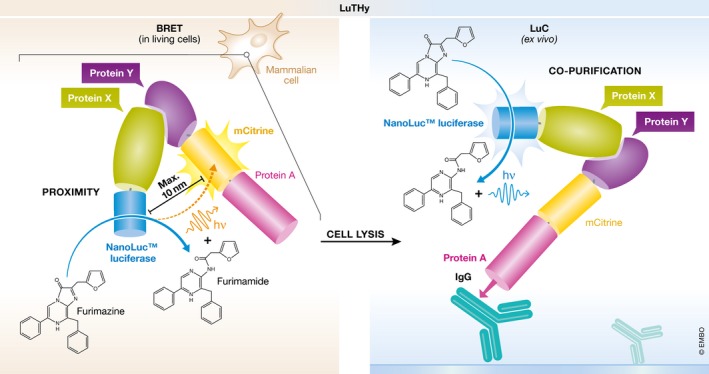
Schematic representation of LuTHy Binary PPIs are detected with a double readout that combines *in vivo* BRET and *ex vivo* luminescence‐based co‐precipitation (LuC). One protein is fused to NanoLuc luciferase and the other protein to protein A (PA) and mCitrine. BRET between the two proteins is measured in the cells expressing the two fusion proteins. Subsequently, the cells are lysed and PA‐mCitrine fusion protein is adsorbed on an IgG‐coated plate, and if the two proteins form a stable complex, luminescence signal can be detected.

Both BRET and a luminescence‐based readout of co‐purification (known as LUMIER) have been previously described and often employed in proteomics (Xu *et al*, 1999; Barrios‐Rodiles *et al*, 2005), so the novel aspect here is their combination in a one‐pot assay. Because these assays are suitably complementary, LuTHy helps to address the commonly assumed weaknesses of the individual assays: tag location and protein complex stability. On the one hand, BRET can be measured in living cells but the assay is highly sensitive to tag location. For example, if proteins interact with their C‐terminal domains, tags in the N‐terminus may not be close enough for a BRET signal. On the other, co‐purification assays require an interaction that stays intact during incubation and wash steps, whereas the tag location is unlikely to affect the signal. These differences sound intuitively reasonable, and the control experiments performed by Trepte *et al* (2018) nicely confirm this notion.

All PPI assays report interactions that do not occur in cells (false positives) and fail to detect interactions that do occur (false negatives), and the key is to find an acceptable balance between the two. To benchmark LuTHy, Trepte *et al* (2018) used a well‐characterized positive reference set of known binary PPIs and a random reference set that consists of random protein pairs that are unlikely to interact with each other (Braun *et al*, 2009). Compared to other existing binary assays, LuTHy performs very well, detecting almost 50% of known interactors with a false‐positive rate of 2.5%. This may sound disappointing, given that an ideal assay would detect *all* known interactions and none of the random protein pairs as interactors. However, a sobering fact is that no currently available PPI assay can detect more than about 40% of well‐characterized binary interactions without dramatically compromising assay specificity (Braun *et al*, 2009; Lievens *et al*, 2014). Using another benchmark set, Trepte *et al* (2018) further show that LuTHy can detect binary interactions covering a range of affinities from micromolar to femtomolar. Thus, the specificity and sensitivity of LuTHy compare very favorably to existing assays.

Protein interaction networks are highly dynamic, changing in response to cellular stimuli, chemical perturbations, splice variants, or pathogenic mutations. Much of the focus of interaction proteomics has recently shifted to characterizing these changes (Sahni *et al*, 2015; Díaz‐Mejía *et al*, 2018). Consequently, any new assay should ideally detect and measure such changes. Using well‐characterized condition‐specific interactions as examples, Trepte *et al* (2018) demonstrate that their assay can quantify interactions regulated by small molecules, environmental conditions (heat shock), and mutations. To address the effects of pathogenic mutations on PPIs, the authors focus on CSPα (DNAJC5), an Hsp70 cofactor localized to synaptic vesicles and mutated in adult‐onset neuronal ceroid lipofuscinosis (ANCL; Nosková *et al*, 2011). They characterize the interactions of wild‐type CSPα and two ANCL‐associated missense variants, L115R and ΔL116, with a set of presynaptic proteins. They identify several novel synaptic interactors for wild‐type CSPα and validate these in primary hippocampal neurons. Interestingly, both mutants showed weaker interactions with membrane‐associated proteins but had stronger interactions with soluble proteins. Consistent with this, immunofluorescence confirmed that the pathogenic CSPα variants are not properly trafficked to the membrane. The functional roles of the novel interactions of wild‐type CSPα or the aberrant interactions of mutant variants are still unknown, but both are clearly worthy of further study.

One of the main strengths of the work by Trepte *et al* (2018) is the thorough validation and benchmarking of LuTHy, making it easy to compare this novel assay to existing ones. Such a comprehensive approach sets a high standard for future PPI assay development. The advantages of the assay are many: It is quantitative, scalable, and compatible with small molecule treatments and diverse conditions, and it uses two independent measurements for detection of interactions. But what might be the weaknesses? One is common to all binary assays: You can only find interactions between proteins that are being tested. Thus, discoveries are limited to those defined by the initial hypothesis or the availability of cDNA clones. Another issue is shared with most high‐throughput methods: Tags and protein fusions can disrupt protein function and interactions, and overexpression can lead to spurious interactions due to the law of mass action. However, Trepte *et al* (2018) nicely address these possibilities, suggesting that they are not a major confounding problem. This is also corroborated by the specificity and sensitivity analysis of LuTHy. Finally, from a practical standpoint, not all laboratories have access to liquid handling instruments, plate washers, plate readers, or large collections of cDNA clones. But this is (hopefully) changing as core facilities are becoming more common and services such as Addgene (addgene.org) have dramatically improved access to reagents.

Of course, the value of any new assay is in the end solely determined by the researchers who adopt it and the biological insights it can provide to the proteomics field and the entire biomedical research community. From a technical perspective, LuTHy is certainly a promising development. Future studies will determine where it will find its place in the expanding toolbox of proteomics.
